# Effect of Mandatory Bicycle Helmet Legislation on Helmet Use and Injury Outcomes: A Propensity Score-Matched Analysis Using Decision Tree and Segmented Regression

**DOI:** 10.3390/jcm15093515

**Published:** 2026-05-04

**Authors:** Hoonsung Park, Maru Kim, Dae-Sang Lee, Tae Hwa Hong, Doo-Hun Kim, Hangjoo Cho

**Affiliations:** 1 Department of Trauma Surgery, Uijeongbu St. Mary’s Hospital, College of Medicine, The Catholic University of Korea, Seoul 06591, Republic of Korea; niedwynn@naver.com (H.P.);; 2 Department of Surgery, Armed Forces Capital Hospital, Seongnam 13574, Republic of Korea

**Keywords:** bicycling, head protective devices, legislation as topic, propensity score, retrospective studies

## Abstract

**Background:** A non-penal bicycle helmet mandate was implemented in the Republic of Korea on 28 September 2018, yet post-implementation epidemiologic evidence remains limited. This study evaluated changes in helmet wearing and injury outcomes before and after the mandate and identified factors associated with helmet non-use. **Methods:** We conducted a retrospective observational study using Korea Trauma Data Bank records from January 2017 to December 2022. After exclusions, 7653 bicycle-injured patients were identified, and 1:1 optimal propensity score matching (caliper 0.10) produced 4798 patients (2399 pairs). Outcomes included helmet use, physiological status (AVPU scale and Revised Trauma Score), Injury Severity Score (ISS), Abbreviated Injury Scale by body region, and mortality. Monthly helmet use trends were analyzed using a regression tree to detect data-driven breakpoints and segmented logistic regression at the legal implementation point (22 months) and the regression-tree-identified split (19.5 months). Multivariable logistic regression identified factors independently associated with helmet non-use. **Results:** Helmet use increased modestly from 17.4% pre-mandate to 21.9% post-mandate (*p* < 0.001). Interrupted time-series analyses showed no immediate level or slope change at either breakpoint, suggesting gradual uptake. ISS values were higher post-mandate, with ISS > 15 increasing from 21.5% to 24.4% (*p* = 0.02). Total mortality rose from 3.9% to 5.3% (*p* = 0.02). Helmet non-use was independently associated with age 80–89 years, female sex, non-Korean nationality, and residence in Jeollabuk-do. **Conclusions:** The mandate was associated with a modest increase in helmet wearing without an abrupt behavioral shift. These findings suggest that legislation without enforcement may have limited impact as a stand-alone measure and should be complemented by targeted education, visitor-focused communication, and region-specific safety measures.

## 1. Introduction

In 2022, there were 12,564 bicycle-related traffic accidents in the Republic of Korea, accounting for 6.4% of all traffic accidents. The number of public bicycle rentals reached 50,844,014 in 2022, a remarkable increase compared with 15,149,155 in 2017 [1]. Several studies have demonstrated that bicycle helmets are effective in protecting against severe head and facial injuries [2,3,4,5,6]. In a recent meta-analysis, bicycle helmet use was associated with a 48% reduction in head injury, 60% in serious head injury, 53% in traumatic brain injury, 23% in facial injury, and a 34% reduction in the number of cyclists killed or seriously injured [7]. This meta-analysis included international studies conducted in diverse settings, including North America, Europe, Oceania, and parts of Asia. However, differences in cycling infrastructure, baseline helmet-wearing culture, and helmet-legislation frameworks may limit direct extrapolation of these pooled estimates to the Republic of Korea.

Under the Republic of Korea’s Road Traffic Act, helmet use has been mandatory since 2011 for drivers and passengers of motorcycles and other motor-driven two-wheeled vehicles, and violations are subject to traffic citations or administrative fines. Helmet use for personal mobility devices (e.g., e-scooters) was not mandated in 2011 but became mandatory in 2021. In contrast, no statutory helmet requirement was applied for bicycle use until 28 September 2018, when an amendment to the Road Traffic Act introduced a mandate for both riders and passengers. Notably, the amendment did not include an enforcement mechanism; therefore, non-compliance did not result in traffic citations or administrative fines [8].

Although studies from the Republic of Korea have described the epidemiology and outcomes of bicycle-related injuries up to 2018 [9,10], to our knowledge, no study has examined the epidemiology or outcomes after the amendment of 28 September 2018 to the Road Traffic Act mandating helmet use for bicyclists, nor has any study compared outcomes before and after the amendment. Accordingly, we analyzed data from the Korea Trauma Data Bank (KTDB) to compare helmet use rates and clinical outcomes before and after the implementation of the amendment (September 2018), determine whether helmet use changed significantly following the law, and identify patient and regional characteristics associated with helmet non-use.

## 2. Materials and Methods

### 2.1. Study Design and Data Collection

In 2012, the Ministry of Health and Welfare of the Republic of Korea initiated a project to establish regional trauma centers for the management of trauma and injury patients. To date, 17 regional trauma centers have been designated and are in operation nationwide. Established in 2013, the KTDB mandates the registration of trauma patient data by institutions participating in the Regional Trauma Center Installation Support Project. The KTDB aims to support research, inform policy development and quality management, and provide the foundation for severe-trauma care systems [11,12,13].

This was a retrospective observational study that used data from the KTDB from 1 January 2017 to 31 December 2022.

### 2.2. Injury Severity Score and Revised Trauma Score

The Injury Severity Score (ISS) is an anatomical measure of trauma severity ranging from 1 to 75. It is derived from the AIS by grouping AIS body regions into six categories (head/neck, face, chest, abdominal/pelvic contents, extremities/pelvic girdle, and external) and summing the squares of the highest AIS grades in the three most severely injured regions [14]. Since the 1980s, an ISS greater than 15 has generally been used to define major trauma and is associated with mortality rates exceeding 20% [15,16,17].

In 1981, Champion et al. first proposed the Trauma Score [18], which was subsequently updated as the Revised Trauma Score (RTS) in 1989 [19]. The RTS is calculated as follows:

Equation (1). Calculation of the weighted RTS:

RTS = (0.9368 × GCS code value) + (0.7326 × SBP code value) + (0.2908 × RR code value)(1)

GCS, Glasgow Coma Scale; SBP, Systolic Blood Pressure; RR, Respiratory rate

The weighted RTS ranges from 0 to 7.8408.

Compared with the Trauma Score, the RTS showed substantially greater reliability for outcome prediction and achieved superior predictive accuracy in patients with severe head injuries [19].

### 2.3. Statistical Analysis

Baseline comparative statistical analyses and propensity score matching were conducted using R software (version 4.5.1; R Foundation for Statistical Computing, Vienna, Austria). Classification and regression tree (CART) modeling, interrupted time-series segmented regression, and figure creation were performed using Python (version 3.11.7; Python Software Foundation). All tests were two-sided (α = 0.05), and effect estimates are presented with 95% confidence intervals. Baseline characteristics between the pre-implementation and post-implementation groups were compared as follows: continuous variables (RTS and ISS) were analyzed with the t test if normally distributed; otherwise, the Mann–Whitney U test was performed; all remaining variables were categorical and were compared using the χ^2^ test or the Fisher exact test, as appropriate. Continuous variables are presented as mean ± SD when normally distributed, and as median and interquartile range when the distribution was non-normal. Normality was assessed using the Shapiro–Wilk test. Homoscedasticity was evaluated using Levene’s test. The variables considered for the multivariable logistic regression were evaluated for multicollinearity. Multicollinearity was assessed using variance inflation factors (VIFs). All VIFs were <5, indicating no evidence of problematic multicollinearity. The Location variable was analyzed after aggregating neighboring provinces into prespecified regional groups.

### 2.4. Propensity Score Matching

To minimize the confounding factors in this retrospective cohort study, we performed propensity score matching. Propensity scores were estimated using logistic regression with the following baseline covariates: age, sex, nationality, insurance, location, regional trauma center. We applied 1:1 optimal matching without replacement using a caliper of 0.10 on the propensity score scale. A caliper of 0.10 on the propensity score scale was chosen after inspecting balance and match yield, which achieved a standardized mean difference (SMD) < 0.10 for most covariates. Of the 7653 eligible patients, 4798 (2399 pairs) were successfully matched. Post-matching covariate balance was assessed using the absolute SMD; an SMD < 0.10 was considered adequate, and an SMD in the range of 0.10–0.20 was considered borderline. Most covariates achieved an adequate balance, with insurance showing a borderline imbalance (SMD = 0.104). The primary analysis was conducted within the matched sample only, without additional post-matching regression adjustment, and the possibility of residual confounding is acknowledged as a study limitation.

### 2.5. Classification and Regression Tree

We trained a CART to identify data-driven split points in helmet use over time. Splits were selected to maximize the reduction in the sum of the squared errors via recursive binary partitioning [20]. The following constraints were imposed: maximum depth = 3, minimum samples to split = 540, and minimum samples per leaf = 180 (scikit-learn parameters). To mitigate overfitting, we applied cost–complexity pruning and selected the final tree using a one-standard-error rule based on 10-fold cross-validated mean squared error. When modeling monthly rates, observations were weighted by the monthly denominator using sample_weight. The model was used to identify data-driven split points that indicated structural changes in the average helmet use rate.

### 2.6. Segmented Regression Analysis

This study employed an interrupted time series-based segmented logistic regression to estimate the effect of the law. The primary breakpoint (n) was prespecified at 22 months, corresponding to the first monthly interval immediately after the mandate took effect, and alternative models were fitted using split points identified by the CART analysis. Where (*t*) denotes months since the start of observation; *I*(*t* ≥ *n*) is an indicator for the post-implementation period; and (*t* − *n*)_+_ = max(0, *t* − *n*) captures the post-implementation change in slope. The model is:

Equation (2). Equation of the segmented regression analysis.

logit(*p_t_*) = *β*_0_ + *β*_1_*t* + *β*_2_*I*(*t* ≥ *n*) + *β*_3_ (*t* − *n*)_+_ (2)

Here, *β*_0_ is the baseline log-odds, *β*_1_ the pre-implementation monthly trend, β_2_ the immediate level change at implementation, and *β*_3_ the change in slope after implementation; thus, the post-implementation monthly trend equals *β*_1_ + *β*_3_. Effects are reported as odds ratios: pre-implementation monthly change = $e^{\beta_{1}}$; immediate change at implementation = $e^{\beta_{2}}$; post-implementation monthly change = $e^{\beta_{1}+\beta_{3}}$. Time was coded in monthly units from January 2017 (1) to December 2022 (72), with month 22 designated as the implementation point (the first month immediately following 28 September 2018).

### 2.7. Reporting Guidelines

This study was conducted in accordance with the Strengthening the Reporting of Observational Studies in Epidemiology (STROBE) guidelines to ensure the completeness and transparency of the observational research.

## 3. Results

**Figure 1:**
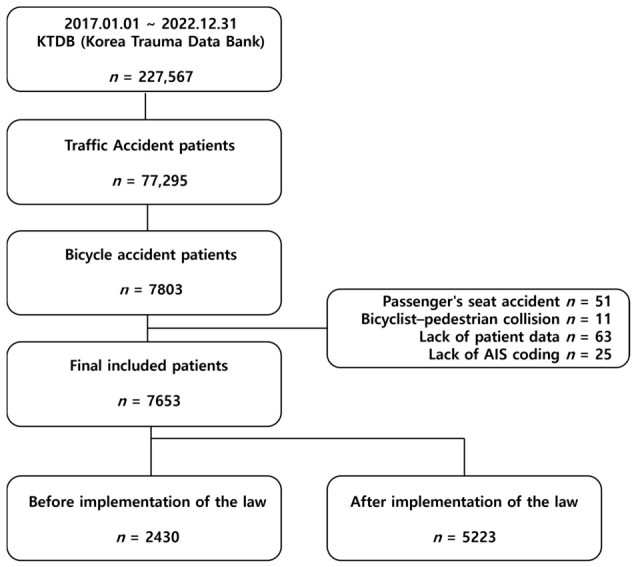
Flow diagram of the study. AIS, Abbreviated Injury Scale.

Figure 1 shows the patient selection process. From the KTDB, 227,567 trauma patients were registered between 1 January 2017 and 31 December 2022, of whom 77,295 sustained traffic-related injuries. Of these, 7803 were bicycle-related. After excluding bicycle passengers (*n* = 51), bicyclist–pedestrian collisions (*n* = 11), records with missing data (*n* = 63), and cases lacking Abbreviated Injury Scale (AIS) coding (*n* = 25), 7653 patients remained for analysis. Patients of all ages and both sexes were included. The cohort was stratified by the implementation date of the bicycle helmet mandate (28 September 2018) into the pre-implementation (*n* = 2430) and post-implementation (*n* = 5223) groups.

Table 1 summarizes the baseline characteristics of the patients. Of the 7653 cases, 2399 pairs were successfully matched. After matching, age, sex, nationality, location, and transfer to a regional trauma center achieved an adequate balance (SMD < 0.10), whereas insurance showed a borderline imbalance (SMD = 0.104). Patients in the 5th decade of life constituted the largest age group in both the pre- and post-implementation groups (17.8% and 18.2%, respectively). Male patients were predominant (82.0% and 82.9%, respectively). The nationals of the Republic of Korea accounted for 97.2% and 97.5% of the pre- and post-implementation groups, respectively. The National Health Insurance was the most frequently used insurance type, followed by Motor Vehicle Insurance. Excluding unknown entries, Gyeonggi-do was the most common location (11.4% and 11.4%), followed by Gyeongsangbuk-do (7.5% and 7.5%). Initial direct transfer to a regional trauma center was less common, with “No” recorded in 55.8% and 51.6% of the cases.

Table 2 presents key outcomes before and after the law in the propensity-score matched cohort, whereas the full outcome comparison, including both unmatched and matched cohorts and detailed outcome categories, is provided in Supplementary Table S1. In both periods, helmet non-use remained more prevalent. In the matched cohort, the helmet-wearing rate increased by 4.5% post-implementation compared with pre-implementation. The proportion of out-of-hospital cardiopulmonary resuscitation did not differ significantly between periods in either cohort, whereas the distribution of AVPU categories differed significantly between pre- and post-implementation in both cohorts. RTS did not differ between periods in either cohort. By contrast, ISS was significantly higher post-implementation in both cohorts. Consistently, the proportion of major trauma (ISS > 15) was significantly higher after implementation in both the unmatched and matched analyses. The distribution of AIS body region also differed significantly between periods, with a lower proportion of head injuries and higher proportion of lower-extremity injuries post-implementation. Dead on arrival, emergency room (ER) mortality, and in-hospital mortality did not differ significantly; however, total mortality (combined mortality of dead on arrival, ER mortality, and in-hospital mortality) differed significantly between periods in both cohorts, showing a 1.4-percentage-point increase after the law.

**Figure 2:**
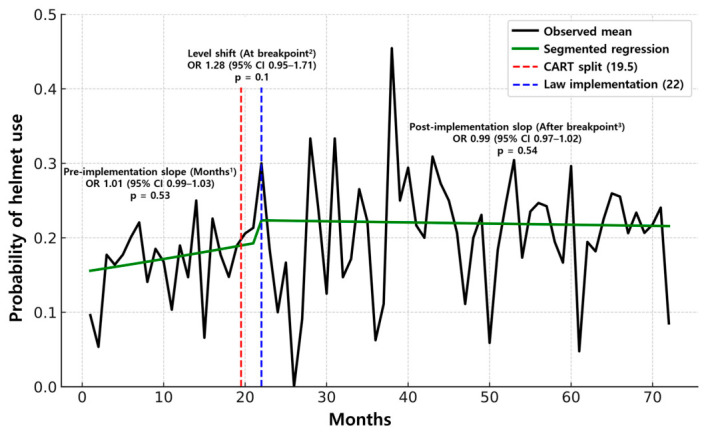
Segmented regression analysis at 22 months. ^1^ Months (*β*_1_): pre-implementation monthly slope. ^2^ At breakpoint (*β*_2_): immediate level change at the breakpoint (detected split 19.5 months or law implementation 22 months). ^3^ After breakpoint (*β*_3_): change in slope after the breakpoint (post-implementation). OR, odds ratio; CI, confidence interval; CART, classification and regression tree.

**Figure 3:**
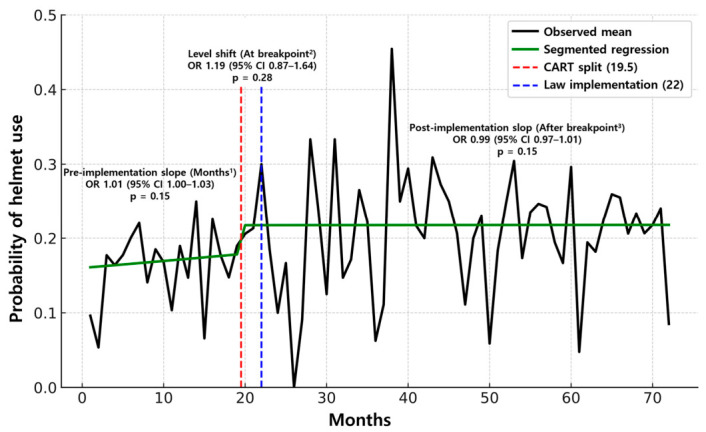
Segmented regression analysis at 19.5 months. ^1^ Months (*β*_1_): pre-implementation monthly slope. ^2^ At breakpoint (*β*_2_): immediate level change at the breakpoint (detected split 19.5 months or law implementation 22 months). ^3^ After breakpoint (*β*_3_): change in slope after the breakpoint (post-implementation). OR, odds ratio; CI, confidence interval; CART, classification and regression tree.

Table 3 summarizes the results of the CART and segmented regression analyses. CART identified an optimal split at 19.5 months. Using 19.5 months as the split point, the prevalence of helmet use increased significantly from 17% to 21.8% (*p* < 0.001). In the segmented regression, at the prespecified implementation point (22 months), neither the immediate level change at the breakpoint nor the post-implementation change in slope after the breakpoint was statistically significant (*p* = 0.10 and *p* = 0.54, respectively; Figure 2). Using the CART-identified breakpoint at 19.5 months yielded the same conclusion: neither the level shift at the breakpoint nor the subsequent slope change after the breakpoint was significant (*p* = 0.28 and *p* = 0.15, respectively; Figure 3).

Table 4 summarizes the key factors associated with helmet non-use, whereas the full univariable and multivariable logistic regression model is provided in Supplementary Table S2. In the multivariable analysis, octogenarians (80–89 years) had higher odds of helmet non-use compared with teenagers (10–19 years) (OR 1.77, 95% CI 1.03–3.06; *p* = 0.04). Relative to Seoul, Jeollabuk-do was associated with higher odds (OR 2.41, 95% CI 1.06–5.48; *p* = 0.04). Non-Korean nationals (compared to nationals of the Republic of Korea) also had higher odds (OR 1.97, 95% CI 1.11–3.52; *p* = 0.02). Female patients had higher odds than male patients (OR 1.74, 95% CI 1.40–2.16; *p* < 0.001).

## 4. Discussion

As shown in Table 1, the insurance variable exhibits a borderline residual imbalance after matching (SMD = 0.104). We used 1:1 optimal matching without replacement with a caliper of 0.10 on the propensity score scale to maximize the covariate balance between the pre- and post-implementation groups. After matching, most covariates achieved adequate balance (SMD < 0.10); only insurance remained borderline (SMD = 0.104), likely driven by sparse categories, such as workers’ compensation insurance. Achieving a stricter target (SMD < 0.10) for such variables would have required shrinking the caliper or imposing exact or fine-balance constraints on insurance, which would increase attrition, reduce the sample size and statistical power, and potentially compromise representativeness. Therefore, in line with our prespecified analysis plan, we conducted primary comparisons within the matched sample only and did not apply additional post-matching regression adjustments. Consequently, a small degree of residual confounding owing to rare categories cannot be fully excluded and is acknowledged as a limitation. Nevertheless, given the methodological guidance that treats SMD < 0.10 as ideal and SMD < 0.20 as acceptable, the residual imbalance observed here (SMD = 0.104) is unlikely to materially bias the effect estimates [21,22].

Although substantial effort has been invested in building a trauma registry through KTDB enrollment, Table 1 shows a high proportion of Unknown in the Location variable, most likely reflecting data entry omissions at the collection point. To minimize sample attrition and selection bias, we retained Unknown as a distinct category and included it in the propensity-score matching procedure. After matching, the distribution of the Location variable was similar between the pre- and post-implementation groups, indicating an adequate balance and a non-differential data-entry error with respect to the implementation period. Nevertheless, a large unknown stratum may hinder the detection of spatial heterogeneity and contribute to residual confounding and greater variance. Therefore, we acknowledge this as a limitation of the present study.

Among the outcomes in Table 2, helmet use increased significantly in the matched cohort post-implementation compared to pre-implementation (17.4% vs. 21.9%; *p* < 0.001). As the propensity score matching emphasizes a cross-sectional pre–post contrast to estimate the average effect, it provides limited insight into the underlying temporal structure of change. To address this, we applied interrupted time-series segmented logistic regressions at two cut points: (1) the prespecified implementation date and (2) the data-driven breakpoint identified by CART, estimating both level and slope changes to evaluate the timing and shape of the response. This complementary strategy preserves the transparency of a simple pre–post comparison while detecting temporal heterogeneity (anticipatory, lagged, or gradual effects), thereby strengthening internal validity and policy interpretability [23].

The observed increase in total mortality despite the modest increase in helmet use should be interpreted cautiously and should not be taken to imply that the mandate or helmet use increased mortality. Possible explanations include regional variation in injury severity and trauma outcomes, differences in patient composition, and unmeasured behavioral changes such as risk compensation. However, such behavioral mechanisms were not directly assessed in this registry-based study and should therefore be regarded as hypotheses rather than definitive explanations.

As shown in Table 3, helmet use increased overall; however, the rise did not present as a sharp level shift or a pronounced slope change at a single time point. Instead, the pattern was consistent with a gradual diffuse change over time. This pattern is consistent with the policy sequence in the Republic of Korea. The Road Traffic Act amendment was passed on 28 March 2018, and after a six-month grace period, the mandate entered into force on 28 September 2018. Notably, the CART-derived breakpoint at 19.5 months precedes the prespecified implementation point (22 months), suggesting anticipatory compliance or the influence of pre-implementation campaigns and media coverage. Although the segmented models anchored at the legal implementation date did not show statistically significant changes in level or in slope, this may indicate either the absence of an immediate discontinuity or an effect size that is too modest to emerge above month-to-month variation. Even so, the pre–post difference in helmet use in the matched cohort (≈+4.5%) is policy-relevant and supports the interpretation of a steady uptake rather than a sudden leap.

Table 4 shows the key factors associated with helmet non-use: being an octogenarian (OR 1.77; *p* = 0.04), resident of Jeollabuk-do (OR 2.41; *p* = 0.04), non-Korean nationality (OR 1.97; *p* = 0.02), and female sex (OR 1.74; *p* < 0.001).

In a nationwide database study from the Republic of Korea, helmet use prevalence peaked at 35 years of age and declined thereafter, falling to <5% among octogenarians [24]. In a separate nationwide analysis of older adults (≥65 years; *n* = 9290) in the Republic of Korea, each 1-year increase in age was associated with lower odds of helmet use (odds, 0.965; *p* < 0.0001) [25]. As shown in Table S3, the helmet use rate in the matched cohort was highest among individuals in their 30s (37.8%) and declined thereafter, reaching a minimum in octogenarians (5.6%). In the unmatched cohort, the peak occurred in individuals in their 40s (36.8%), with a similar decline of 4.9% among octogenarians. Taken together with prior nationwide evidence from the Republic of Korea, these findings suggest that lower helmet use among older adults is a consistent, country-specific pattern. Following the national plan to promote bicycle use in the Republic of Korea, most public education and intervention programs related to helmet use have primarily targeted adolescent and school-enrolled populations [26]. Despite a decade of such programming, older adults who had been cycling before the rollout were unlikely to be directly targeted, making behavioral change more difficult. The observed disparity in helmet use rates between the younger and older cohorts may also reflect the country’s rapid transition to a bicycle-friendly society [24]. Therefore, targeted education and outreach are warranted for elderly populations.

From 2015 to 2023, the bicycle fatality rate in Jeollabuk-do exceeded the annual national mean each year, and the highest nationwide cyclist-at-fault fatality rates (7.0%, 9.9%, and 6.0%) were recorded in 2021–2023 [27]. After direct age and sex standardization, the case-fatality rate (CFR) in Jeollabuk-do was 16.5%, which is 3.7 times higher than the CFR of all other regions combined (4.5%; Table S4). Jeollabuk-do also had a higher proportion of elder patients (Table S5) (≥60 years: 61.4% vs. 42.0%; ≥70 years: 41.8% vs. 23.3%) and, even within the same age strata, the CFR remained higher: <60 years: 15.3% vs. 1.7% (*p* < 0.001); 60–69 years: 16.7% vs. 5.4% (*p* = 0.02) (Table S6). Indicators of injury severity and mortality were also elevated: ISS > 15, 43.1% vs. 24.7% (*p* < 0.001); emergency room mortality, 3.3% vs. 0.5% (*p* < 0.01); and in-hospital mortality, 11.8% vs. 3.4% (*p* < 0.001) (Table S7). Taken together, during the study period, Jeollabuk-do exhibited an age- and sex-standardized CFR 3.7-fold higher than that of the rest of the Republic of Korea, and even accounting for its older case mix, the within-age fatality remained higher. The concurrent elevation in trauma severity shown as ISS > 15 and death endpoints, such as ER and in-hospital mortality, suggests that factors beyond population aging—such as helmet-wear compliance, road environment, prehospital transport, hospital access, and trauma system capacity—may be contributing factors. This interpretation is consistent with regional trauma treatment performance (e.g., the 2021 preventable trauma death rate ranking Gwangju/Jeolla-do/Jeju lowest nationwide), which points to operational challenges, including staffing shortages in Jeollabuk-do regional trauma centers.

Regarding helmet use among non-Korean nationals, bike-sharing users have been reported to have low helmet wearing rates [28,29]. In addition, official English-language guidance for visitors such as VisitKOREA describes helmet use as “recommended” which foreign short-term visitors and tourists may not interpret as a legal requirement in the Republic of Korea [30]. Therefore, targeted outreach to inform inbound visitors that helmet use is mandatory is warranted.

Women engage in short utility cycling more often than men, which may have contributed to their lower helmet use. User-centered designs that accommodate hair volume and improve comfort have been suggested [31]. However, some studies have reported higher odds of helmet use among women than men [32], indicating that sex differences likely vary by setting and region. Targeted remediation of these issues may reduce helmet non-use and improve safety among bicycle riders.

## 5. Limitations

This study has several limitations. First, although propensity-score matching improved the balance between the pre- and post-implementation groups, insurance showed borderline residual imbalance after matching (SMD = 0.104). Because the primary comparisons were conducted within the matched sample without additional post-matching regression adjustment, a small degree of residual confounding owing to rare categories cannot be fully excluded. Second, the Location variable included a high proportion of Unknown entries, accounting for 37.1% of the matched cohort, most likely reflecting data-entry omissions at the collection point. Although the Unknown category was retained and balanced between groups after matching, this large unknown stratum may have hindered the detection of spatial heterogeneity and contributed to residual confounding and greater variance. Third, propensity-score matching provides a cross-sectional pre–post contrast and has limited ability to capture the temporal structure of change; therefore, the findings from the matched comparison should be interpreted together with the CART and segmented regression analyses. Fourth, the study used KTDB data up to 31 December 2022, which may not fully reflect more recent changes in bicycle use, helmet-wearing behavior, or trauma-system conditions. This reflects the delay in availability of national registry data for research use and should be considered when interpreting the current policy relevance of the findings.

## 6. Conclusions

Using KTDB data, we found that helmet use increased after the 2018 mandate in matched comparisons (from 17.4% to 21.9%, *p* < 0.001); however, segmented regression detected no significant step or slope change at 22 months or at the CART-derived breakpoint, consistent with diffuse, anticipatory adoption. Multivariable analyses identified factors associated with helmet non-use, including age 80–89 years (OR 1.77, *p* = 0.04), female sex (OR 1.74, *p* < 0.001), non-Korean nationality (OR 1.97, *p* = 0.02), and Jeollabuk-do (OR 2.41, *p* = 0.04). These findings suggest that non-penal helmet legislation may have limited impact as a stand-alone measure and should be complemented by targeted education, visitor-focused communication, and region-specific safety measures. Our results refine the evidence base by combining matching, CART, and interrupted time series within a single framework to separate the average pre/post differences from the temporal structure.

## Figures and Tables

**Table 1:** Patient characteristics.

	Pre-Matching			Post-Matching		
	Pre-Implementation	Post-Implementation	SMD	Pre-Implementation	Post-Implementation	SMD
Number	2430	5223		2399	2399	
Age			0.15			0.05
1–9	142 (5.8)	239 (4.6)		139 (5.8)	125 (5.2)	
10–19	330 (13.6)	608 (11.6)		323 (13.5)	315 (13.1)	
20–29	148 (6.1)	245 (4.7)		148 (6.2)	148 (6.2)	
30–39	172 (7.1)	313 (6.0)		170 (7.1)	163 (6.8)	
40–49	246 (10.1)	539 (10.3)		244 (10.2)	250 (10.4)	
50–59	428 (17.6)	997 (19.1)		426 (17.8)	437 (18.2)	
60–69	406 (16.7)	1028 (19.7)		400 (16.7)	410 (17.1)	
70–79	394 (16.2)	820 (15.7)		385 (16.0)	380 (15.8)	
80–89	154 (6.3)	415 (7.9)		154 (6.4)	165 (6.9)	
over 90	10 (0.4)	19 (0.4)		10 (0.4)	6 (0.3)	
Sex			0.04			0.03
Male	1992 (82.0)	4206 (80.5)		1966 (82.0)	1989 (82.9)	
Female	438 (18.0)	1017 (19.5)		433 (18.0)	410 (17.1)	
Nationality			0.07			0.02
Korean	2363 (97.2)	5011 (95.9)		2333 (97.2)	2339 (97.5)	
Non-Korean	67 (2.8)	212 (4.1)		66 (2.8)	60 (2.5)	
Insurance			0.11			0.10 ^†^
NHI	1463 (60.2)	3276 (62.7)		1454 (60.6)	1455 (60.7)	
MVI	842 (34.7)	1665 (31.9)		822 (34.3)	866 (36.1)	
MAP type 1	50 (2.1)	147 (2.8)		50 (2.1)	39 (1.6)	
MAP type 2	14 (0.6)	32 (0.6)		14 (0.6)	7 (0.3)	
WCI	2 (0.1)	17 (0.3)		2 (0.1)	1 (0.0)	
Etc ^1^	59 (2.4)	86 (1.6)		57 (2.4)	31 (1.3)	
Location			0.3			<0.001
Busan	50 (2.1)	107 (2.0)		50 (2.1)	50 (2.1)	
Chungcheongbuk-do	112 (4.6)	138 (2.6)		112 (4.7)	112 (4.7)	
Chungcheongnam-do	68 (2.8)	171 (3.3)		68 (2.8)	68 (2.8)	
Daegu	91 (3.7)	148 (2.8)		91 (3.8)	91 (3.8)	
Daejeon	129 (5.3)	209 (4.0)		129 (5.4)	129 (5.4)	
Gangwon-do	83 (3.4)	197 (3.8)		83 (3.5)	83 (3.5)	
Gwangju	4 (0.2)	4 (0.1)		4 (0.2)	4 (0.2)	
Gyeonggi-do	274 (11.3)	730 (14.0)		274 (11.4)	274 (11.4)	
Gyeongsangbuk-do	180 (7.4)	356 (6.8)		180 (7.5)	180 (7.5)	
Gyeongsangnam-do	31 (1.3)	106 (2.0)		31 (1.3)	31 (1.3)	
Incheon	5 (0.2)	11 (0.2)		5 (0.2)	5 (0.2)	
Jeju	27 (1.1)	169 (3.2)		27 (1.1)	27 (1.1)	
Jeollabuk-do	92 (3.8)	61 (1.2)		61 (2.5)	61 (2.5)	
Jeollanam-do	109 (4.5)	288 (5.5)		109 (4.5)	109 (4.5)	
Sejong	26 (1.1)	34 (0.7)		26 (1.1)	26 (1.1)	
Seoul	129 (5.3)	224 (4.3)		129 (5.4)	129 (5.4)	
Ulsan	129 (5.3)	213 (4.1)		129 (5.4)	129 (5.4)	
Unknown ^2^	891 (36.7)	2057 (39.4)		891 (37.1)	891 (37.1)	
Regional Trauma Center ^3^			0.3			0.09
YES	1060 (43.6)	3054 (58.5)		1060 (44.2)	1162 (48.4)	
NO	1370 (56.4)	2169 (41.5)		1339 (55.8)	1237 (51.6)	

SMDs (absolute values) are shown to two decimals; thresholds: < 0.10 balanced, 0.10–0.20 borderline, ≥ 0.20 imbalanced. ^†^ Insurance: SMD = 0.104 (borderline). ^1^ Traveler’s insurance, patriots, and veterans. ^2^ Not specified in the record. ^3^ YES; Initially transported directly to a regional trauma center, NO; Initially transported to non-regional trauma center. SMD, Standardized Mean Difference; NHI, National Health Insurance; MVI, Motor Vehicle Insurance; MAP, Medical Aid Program; WCI, Workers’ Compensation Insurance.

**Table 2:** Key outcomes before and after implementation of the bicycle helmet mandate in the propensity-score matched cohort.

Outcome	Pre-Implementation (*n* = 2399)	Post-Implementation (*n* = 2399)	*p*-Value
Helmet use			<0.001
YES	418 (17.4)	525 (21.9)	
NO	1981 (82.6)	1874 (78.1)	
Out-of-hospital CPR			0.37
YES	20 (0.8)	26 (1.1)	
NO	2379 (99.2)	2373 (98.9)	
GCS initial ^†^	15.00 [15.00, 15.00]	15.00 [15.00, 15.00]	0.01
RTS (weighted)	7.84 [7.84, 7.84]	7.84 [7.84, 7.84]	0.2
ISS ^‡^	9.00 [4.00, 14.00]	9.00 [4.00, 14.00]	<0.001
ISS > 15			0.02
YES	515 (21.5)	585 (24.4)	
NO	1884 (78.5)	1814 (75.6)	
DOA			0.57
YES	12 (0.5)	16 (0.7)	
NO	2387 (99.5)	2383 (99.3)	
ER mortality			0.12
YES	6 (0.3)	14 (0.6)	
NO	2393 (99.7)	2385 (99.4)	
In-hospital mortality			0.09
YES	75 (3.1)	98 (4.1)	
NO	2324 (96.9)	2301 (95.9)	
Total mortality ^1^			0.02
YES	93 (3.9)	128 (5.3)	
NO	2306 (96.1)	2271 (94.7)	

^†^ Non-normal distribution by Shapiro–Wilk; between-group comparison by Mann–Whitney U. Mean ± SD provided for context: 14.34 ± 2.24 (pre-implementation) vs. 14.19 ± 2.43 (post-implementation). ^‡^ Non-normal distribution by Shapiro–Wilk; between-group comparison by Mann–Whitney U. Mean ± SD provided for context: 9.85 ± 8.51 (pre-implementation) vs. 10.94 ± 9.49 (post-implementation). ^1^ The sum of DOA, ER mortality, and in-hospital mortality. CPR, Cardiopulmonary resuscitation; GCS, Glasgow coma scale; RTS, Revised trauma score; ISS, Injury severity scale; DOA, Dead on arrival; ER, Emergency room.

**Table 3:** Results of CART and segmented regression analysis in propensity-score matched cohort.

Classification and Regression Tree				Segmented Regression Analysis							
Detected Split = 19.5 Months				19.5 Months ^†^				22 Months ^‡^			
Time	Total Number	Helmet User (%)	*p*-Value	Variable	Estimate (*β*)	OR (95% CI)	*p*-Value	Variable	Estimate (*β*)	OR (95% CI)	*p*-Value
Before 19.5 months	2115	359 (17%)	<0.001	Months ^1^	0.013	1.01 (1.00–1.03)	0.15	Months ^1^	0.007	1.01 (0.99–1.03)	0.53
				At breakpoint ^2^	0.175	1.19 (0.87–1.64)	0.28	At breakpoint ^2^	0.244	1.28 (0.95–1.71)	0.1
After 19.5 months	2683	584 (21.8%)		After breakpoint ^3^	−0.014	0.99 (0.97–1.01)	0.15	After breakpoint ^3^	−0.007	0.99 (0.97–1.02)	0.54

^†^ The time of the detected split by CART. ^‡^ The time of law implementation. ^1^ Months (*β*_1_): pre-implementation monthly slope. ^2^ At breakpoint (*β*_2_): immediate level change at the breakpoint (detected split 19.5 months or law implementation 22 months). ^3^ After breakpoint (*β*_3_): change in slope after the breakpoint (post-implementation). CART, classification and regression tree; CI, confidence interval; OR, odds ratio.

**Table 4:** Key factors associated with helmet non-use in the propensity-score matched cohort.

Factor	Reference	Adjusted OR (95% CI)	*p*-Value
Age 80 to 89 years	Age 10 to 19 years	1.77 (1.03–3.06)	0.04
Jeollabuk-do	Seoul	2.41 (1.06–5.48)	0.04
Non-Korean	Korean	1.97 (1.11–3.52)	0.02
Female	Male	1.74 (1.40–2.16)	<0.001

## Data Availability

The data analyzed in this study were provided by the National Emergency Medical Center (NEMC), Republic of Korea. Due to restrictions related to institutional policies, the datasets are not publicly available. Access to the Korea Trauma Data Bank requires application and approval from the NEMC and the appropriate Institutional Review Board. All data were fully anonymized before being released to the investigators.

## Supplementary Materials

The following supporting information can be downloaded at: https://www.mdpi.com/article/10.3390/jcm15093515/s1, Table S1: Outcomes before and after the law implementation.; Table S2: Factors associated with helmet non-use in propensity-score matched cohort. Table S3: Age-specific helmet use rates before and after matching (study dataset).; Table S4: Age and sex-standardized CFR by location.; Table S5: Age-specific patient distribution and proportions of elderly patients in Jeollabuk-do and other regions.; Table S6: Comparison of age-specific case-fatality rates between Jeollabuk-do and other regions.; Table S7: Comparison of severe trauma (ISS > 15) patients and ER/in-hospital deaths between Jeollabuk-do and other regions.

## Abbreviations

The following abbreviations are used in this manuscript:

AIS	Abbreviated Injury Scale
AVPU	Alert, Verbal, Pain, Unresponsive
CART	Classification and Regression Tree
CFR	Case-Fatality Rate
CI	Confidence Interval
CPR	Cardiopulmonary Resuscitation
DOA	Dead on Arrival
ER	Emergency Room
GCS	Glasgow Coma Scale
IRB	Institutional Review Board
ISS	Injury Severity Score
KTDB	Korea Trauma Data Bank
MAP	Medical Aid Program
MVI	Motor Vehicle Insurance
NEMC	National Emergency Medical Center
NHI	National Health Insurance
OR	Odds Ratio
RR	Respiratory Rate
RTS	Revised Trauma Score
SBP	Systolic Blood Pressure
SD	Standard Deviation
SMD	Standardized Mean Difference
VIF	Variance Inflation Factor
WCI	Workers’ Compensation Insurance
