# Supplementary material for: Effect of Mandatory Bicycle Helmet Legislation on Helmet Use and Injury Outcomes: A Propensity Score-Matched Analysis Using Decision Tree and Segmented Regression
Source: J Clin Med. 2026 May 4;15(9):3515. doi: 10.3390/jcm15093515 (PMC13164154; doi:10.3390/jcm15093515)
Supplement: Supplementary file 1 [file jcm-15-03515-s001.zip › jcm-4282144-supplementary.pdf]

Supplementary Materials

Table S1. Outcomes before and after the law implementation.

Pre-matching				Post-matching			
Group	Pre-implementation	Post-implementation	*p*-Value	Group	Pre-implementation	Post-implementation	*p*-Value
Number	2430	5223		Number	2399	2399	
Helmet use			<0.001	Helmet use			<0.001
YES	422 (17.4)	1133 (21.7)		YES	418 (17.4)	525 (21.9)	
NO	2008 (82.6)	4090 (78.3)		NO	1981 (82.6)	1874 (78.1)	
Out-of-hospital CPR			0.19	Out-of-hospital CPR			0.37
YES	20 (0.8)	60 (1.1)		YES	20 (0.8)	26 (1.1)	
NO	2410 (99.2)	5163 (98.9)		NO	2379 (99.2)	2373 (98.9)	
AVPU[1] initial			<0.01	AVPU initial			0.03
A	2120 (87.2)	4390 (84.1)		A	2089 (87.1)	2031 (84.7)	
V	140 (5.8)	355 (6.8)		V	140 (5.8)	145 (6.0)	
P	114 (4.7)	290 (5.6)		P	114 (4.8)	141 (5.9)	
U	56 (2.3)	188 (3.6)		U	56 (2.3)	82 (3.4)	
GCS initial	14.35 ± 2.23	14.18 ± 2.45	<0.01	GCS initial[†]	15.00 [15.00, 15.00]	15.00 [15.00, 15.00]	0.01
RTS (weighted)	7.84 [7.84, 7.84]	7.84 [7.84, 7.84]	0.03	RTS (weighted)	7.84 [7.84, 7.84]	7.84 [7.84, 7.84]	0.2
ISS	9.84 ± 8.48	11.36 ± 9.52	<0.001	ISS[‡]	9.00 [4.00, 14.00]	9.00 [4.00, 14.00]	<0.001
ISS > 15			<0.001	ISS > 15			0.02
YES	519 (21.4)	1396 (26.7)		YES	515 (21.5)	585 (24.4)	
NO	1911 (78.6)	3827 (73.3)		NO	1884 (78.5)	1814 (75.6)	
AIS body region			0.01	AIS body region			0.01
Head	532 (21.9)	1081 (20.7)		Head	526 (21.9)	466 (19.4)	
Face	339 (14.0)	767 (14.7)		Face	337 (14.0)	360 (15.0)	
Neck	10 (0.4)	9 (0.2)		Neck	9 (0.4)	4 (0.2)	
Thorax	218 (9.0)	558 (10.7)		Thorax	216 (9.0)	224 (9.3)	
Abdomen and Pelvis	141 (5.8)	283 (5.4)		Abdomen and Pelvis	139 (5.8)	113 (4.7)	
Spine	193 (7.9)	367 (7.0)		Spine	191 (8.0)	161 (6.7)	
Upper extremity	575 (23.7)	1120 (21.4)		Upper extremity	568 (23.7)	563 (23.5)	
Lower extremity	386 (15.9)	943 (18.1)		Lower extremity	377 (15.7)	461 (19.2)	
External and others	36 (1.5)	95 (1.8)		External and others	36 (1.5)	47 (2.0)	
DOA			0.5	DOA			0.57

YES	12 (0.5)	34 (0.7)		YES	12 (0.5)	16 (0.7)	
NO	2418 (99.5)	5189 (99.3)		NO	2387 (99.5)	2383 (99.3)	
ER mortality			0.02	ER mortality			0.12
YES	6 (0.2)	38 (0.7)		YES	6 (0.3)	14 (0.6)	
NO	2424 (99.8)	5185 (99.3)		NO	2393 (99.7)	2385 (99.4)	
In-hospital mortality			0.14	In-hospital mortality			0.09
YES	75 (3.1)	198 (3.8)		YES	75 (3.1)	98 (4.1)	
NO	2355 (96.9)	5025 (96.2)		NO	2324 (96.9)	2301 (95.9)	
Total mortality[2]			0.01	Total mortality			0.02
YES	93 (3.8)	270 (5.2)		YES	93 (3.9)	128 (5.3)	
NO	2337 (96.2)	4953 (94.8)		NO	2306 (96.1)	2271 (94.7)	

†Non-normal distribution by Shapiro–Wilk; between-group comparison by Mann–Whitney U. Mean ± SD provided for context: 14.34 ± 2.24 (pre-implementation) vs 14.19 ± 2.43 (post-implementation). ‡Non-normal distribution by Shapiro–Wilk; between-group comparison by Mann–Whitney U. Mean ± SD provided for context: 9.85 ± 8.51 (pre-implementation) vs 10.94 ± 9.49 (post-implementation). [1]A, alert; V, verbal; P, pain; U, unresponsive. [2]The sum of DOA, ER mortality, and in-hospital mortality. CPR, Cardiopulmonary resuscitation; GCS, Glasgow coma scale; RTS, Revised trauma score; ISS, Injury severity scale; AIS, Abbreviated Injury Scale; DOA, Dead on arrival; ER, Emergency room.

Table S2. Factors associated with helmet non-use in propensity-score matched cohort.

Post-matching (*n* = 4798)	Reference	Univariable		Multivariable	
		Crude OR (95% CI)	Crude *p*-Value	Adjusted OR (95% CI)	Adjusted *p*-Value
Law implementation status					
Post	Pre	0.75 (0.65–0.87)	<0.001	0.74 (0.63–0.86)	<0.001
Age					
1 to 9	10 to 19	1.15 (0.73–1.8)	0.56		
20 to 29		0.59 (0.41–0.86)	<0.01	0.53 (0.36–0.78)	<0.01
30 to 39		0.23 (0.17–0.32)	<0.001	0.21 (0.15–0.30)	<0.001
40 to 49		0.29 (0.21–0.39)	<0.001	0.26 (0.19–0.36)	<0.001
50 to 59		0.32 (0.24–0.42)	<0.001	0.27 (0.20–0.36)	<0.001
60 to 69		0.63 (0.47–0.85)	<0.01	0.47 (0.34–0.64)	<0.001
70 to 79		1.72 (1.21–2.46)	<0.01	1.24 (0.86–1.80)	0.25
80 to 89		2.36 (1.39–4.02)	<0.01	1.77 (1.03–3.06)	0.04
Over 90		2.12 (0.28–16.27)	0.47		
AIS body region					
Face	Head	0.47 (0.35–0.62)	<0.001	0.71 (0.52–0.97)	0.03
Neck		1.18 (0.15–9.2)	0.87		
Thorax		0.33 (0.24–0.45)	<0.001	0.48 (0.35–0.68)	<0.001
Abdomen and pelvis		0.44 (0.3–0.65)	<0.001	0.55 (0.36–0.83)	<0.01
Spine		0.3 (0.22–0.42)	<0.001	0.45 (0.32–0.64)	<0.001
Upper extremity		0.26 (0.2–0.34)	<0.001	0.43 (0.32–0.57)	<0.001
Lower extremity		0.39 (0.3–0.52)	<0.001	0.52 (0.39–0.71)	<0.001
External and others		0.38 (0.22–0.68)	<0.01	0.5 (0.27–0.94)	0.03
ER AVPU[1]					
V	A	2.32 (1.58–3.41)	<0.001	1.43 (0.94–2.19)	0.09
P		2.89 (1.86–4.51)	<0.001	1.22 (0.73–2.03)	0.44
U		5.11 (2.38–10.98)	<0.001	1.53 (0.61–3.86)	0.37
Insurance					
ETC[2]	MVI	0.68 (0.38–1.20)	0.18		
MAP type 1		0.99 (0.52–1.88)	0.97		

MAP type 2		2.78 (0.37–20.82)	0.32		
NHI		0.43 (0.36–0.51)	<0.001	0.59 (0.49–0.71)	<0.001
WCI		0.28 (0.03–3.08)	0.3		
ISS	per 1-unit increase	1.03 (1.02–1.04)	<0.001	1.01 (1.00–1.02)	0.19
Location					
Busan/Ulsan/Gyeongsangnam-do	Seoul	0.87 (0.6–1.26)	0.47		
Chungcheongbuk-do		1.23 (0.79–1.92)	0.37		
Daegu		1.78 (1.07–2.98)	0.03	1.29 (0.74–2.25)	0.37
Daejeon/Sejong/Chungcheongnam-do		0.87 (0.6–1.25)	0.44		
Gwangju/Jeollanam-do		1.28 (0.82–2.0)	0.28		
Gyeonggi-do/Incheon		1.6 (1.1–2.33)	0.01	1.48 (0.99–2.23)	0.06
Jeju		0.67 (0.35–1.3)	0.24		
Jeollabuk-do		4.04 (1.86–8.77)	<0.001	2.41 (1.06–5.48)	0.04
Gangwon-do		0.84 (0.53–1.32)	0.45		
Gyeongsangbuk-do		1.31 (0.88–1.96)	0.18		
Unknown		1.16 (0.84–1.59)	0.37		
Nationality					
Non-Korean	Korean	1.7 (1.0–2.89)	0.049	1.97 (1.11–3.52)	0.02
Sex					
Female	Male	1.45 (1.18–1.78)	<0.001	1.74 (1.40–2.16)	<0.001
Regional Trauma Center					
No	Yes	0.82 (0.71–0.94)	0.01	1.06 (0.88–1.29)	0.53
Total mortality[3]					
Death	Survive	6.84 (3.36–13.9)	<0.001	2.26 (0.97–5.27)	0.06

[1]A, alert; V, verbal; P, pain; U, unresponsive. [2]Traveler's insurance, patriots, and veterans. [3]The sum of DOA, ER mortality, and in-hospital mortality. AIS, Abbreviated Injury Scale; ER, Emergency room; MAP, Medical Aid Program; NHI, National Health Insurance; WCI, Workers' Compensation Insurance; MVI, Motor Vehicle Insurance; ISS, Injury severity scale.

Table S3. Age-specific helmet use rates before and after matching (study dataset).

**Age**	**Helmet use number / Total number (%)**	
	1555 / 7653 (20.3%)	943 / 4798 (19.7%)
	**Pre-matching**	**Post-matching**
	***n* (%)**	***n* (%)**
1–9	41 (10.8%)	29 (11.0%)
10–19	123 (13.1%)	79 (12.4%)
20–29	81 (20.6%)	57 (19.3%)
30–39	174 (35.9%)	126 (37.8%)
40–49	289 (36.8%)	163 (33.0%)
50–59	434 (30.5%)	264 (30.6%)
60–69	287 (20.0%)	148 (18.3%)
70–79	97 (8.0%)	58 (7.6%)
80–89	28 (4.9%)	18 (5.6%)
over 90	1 (3.4%)	1 (6.2%)

Table S4. Age and sex-standardized CFR by location.

Location	Cases	Deaths	Crude CFR<br>% (95% CI)	Standardized CFR<br>% (95% CI)
Busan	157	29	18.5 (12.7–25.4)	20.0 (15.1–24.8)
Gwangju	8	2	25.0 (3.2–65.1)	21.1 (11.4–30.9)
Jeollabuk-do	153	26	17.0 (11.4–23.9)	16.5 (10.3–22.7)
Chungcheongnam-do	239	31	13.0 (9.0–17.9)	12.3 (8.6–15.9)
Sejong	60	5	8.3 (2.8–18.4)	10.3 (2.3–18.3)
Daegu	239	27	11.3 (7.6–16.0)	9.8 (6.4–13.3)
Gyeonggi-do	1004	48	4.8 (3.5–6.3)	5.1 (3.7–6.4)
Chungcheongbuk-do	250	14	5.6 (3.1–9.2)	5.0 (2.6–7.5)
Gyeongsangnam-do	137	5	3.6 (1.2–8.3)	4.8 (0.3–9.2)
Incheon	16	1	6.2 (0.2–30.2)	5.5 (0.0–11.2)
Jeollanam-do	397	15	3.8 (2.1–6.2)	4.1 (2.1–6.1)
Gyeongsangbuk-do	536	25	4.7 (3.0–6.8)	3.6 (2.2–5.0)
Daejeon	338	13	3.8 (2.1–6.5)	3.6 (1.6–5.5)
Unknown	2947	92	3.1 (2.5–3.8)	3.3 (2.7–4.0)
Gangwon-do	280	9	3.2 (1.5–6.0)	3.1 (1.2–5.0)
Seoul	353	10	2.8 (1.4–5.1)	3.1 (1.2–4.9)
Ulsan	342	9	2.6 (1.2–4.9)	2.9 (0.9–4.9)
Jeju	196	2	1.0 (0.1–3.6)	1.3 (0.0–3.3)
Etc	1	0	0.0 (0.0–97.5)	4.5 (4.1–5.0)
Except Jeollabuk-do	7500	337	4.5 (4.0–5.0)	4.5 (4.1–5.0)

CFR, Case-Fatality Rate (deaths / (deaths + cases) x 100)

Table S5. Age-specific patient distribution and proportions of elderly patients in Jeollabuk-do and other regions.

Age	Total Number		*p*-Value
	153	7500	
	**Jeollabuk-do (%)**	**Others (%)**	
1–9	2	5	<0.001
10–19	11.8	12.3	
20–29	3.9	5.2	
30–39	3.9	6.4	
40–49	6.5	10.3	
50–59	10.5	18.8	
60–69	19.6	18.7	
70–79	26.8	15.6	
80–89	14.4	7.3	
over 90	0.7	0.4	
Age ≥60 (%)	61.4	42.0	<0.001
Age ≥70 (%)	41.8	23.3	<0.001

Table S6. Comparison of age-specific case-fatality rates between Jeollabuk-do and other regions.

Ages	Jeollabuk-do		Others		*p*-Value
	CFR<br>% (95% CI)	Deaths /<br>Total number	CFR<br>% (95% CI)	Deaths /<br>Total number	
<60	15.3<br>(8.2–26.5)	9 / 59	1.7<br>(1.3–2.1)	72 / 4348	<0.001
60–69	16.7<br>(7.3–33.6)	5 / 30	5.4<br>(4.3–6.7)	76 / 1404	0.02
≥70	18.8<br>(11.1–30.0)	12 / 64	10.8<br>(9.4–12.4)	189 / 1748	0.06

CFR, Case-Fatality Rate (deaths / (deaths + cases) x 100)

Table S7. Comparison of severe trauma (ISS >15) patients and ER/in-hospital deaths between Jeollabuk-do and other regions.

Variable	Jeollabuk-do<br>Cases / Total	Others<br>Cases / Total	*p*-Value
ISS >15 among all	66 / 153 (43.1%)	1849 / 7500 (24.7%)	<0.001
ISS >15 among mortality	24 / 26 (92.3%)	285 / 337 (84.6%)	0.4
DOA	3 / 153 (2%)	43 / 7500 (0.6%)	0.06
ER mortality	5 / 153 (3.3%)	39 / 7500 (0.5%)	<0.01
In-hospital mortality	18 / 153 (11.8%)	255 / 7500 (3.4%)	<0.001

ISS, Injury severity scale; DOA, Dead on arrival; ER, Emergency room
